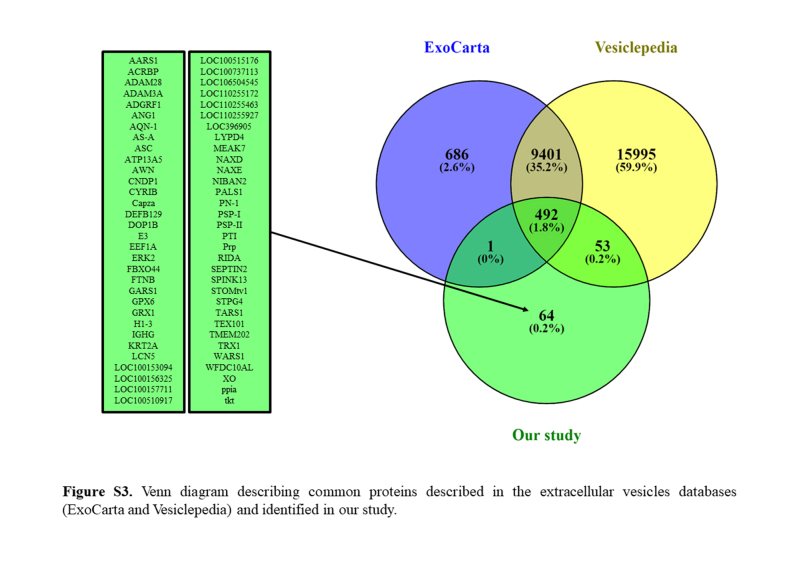# The Proteome of Large or Small Extracellular Vesicles in Pig Seminal Plasma Differs, Defining Sources and Biological Functions

**DOI:** 10.1016/j.mcpro.2023.100514

**Published:** 2023-02-14

**Authors:** Isabel Barranco, Christian M. Sanchez-López, Diego Bucci, Alberto Alvarez-Barrientos, Heriberto Rodriguez-Martinez, Antonio Marcilla, Jordi Roca

**Affiliations:** 1 Department of Veterinary Medical Sciences, University of Bologna, Ozzano dell’Emilia, Bologna, Italy; 2 Àrea de Parasitologia, Departament de Farmàcia i Tecnologia Farmacèutica i Parasitologia, Universitat de València, Burjassot, Valencia, Spain; 3 Joint Research Unit on Endocrinology, Nutrition and Clinical Dietetics, Health Research Institute La Fe-Universitat de València, Valencia, Spain; 4 Servicio de Técnicas Aplicadas a las Biociencias, Universidad de Extremadura, Badajoz, Spain; 5 Department of Biomedical & Clinical Sciences (BKV), University of Linköping, Linköping, Sweden; 6 Department of Medicine and Animal Surgery, Faculty of Veterinary Science, University of Murcia, Murcia, Spain

**Keywords:** seminal plasma, extracellular vesicles, subsets, proteomics, pig, AI, artificial insemination, DLS, dynamic light scattering, EVs, extracellular vesicles, FDR, false discovery rate, FSC, forward scatter, GO, gene ontology, L-EVs, large extracellular vesicles, LC-MS/MS, liquid chromatography–tandem mass spectrometry, MS, mass spectrometry, MISEV 2018, minimal information for studies of extracellular vesicles 2018, NTA, nanoparticle tracking analysis, PBS, phosphate buffered saline, RT, room temperature, sEVs, seminal extracellular vesicles, S-EVs, small extracellular vesicles, SEC, size exclusion chromatography, SP, seminal plasma, SSC, side scatter, SWATH-MS, sequential window acquisition of all theoretical mass spectra, TEM, transmission electron microscopy, TOF, time of flight, XIC, extracted ion chromatogram

## Abstract

Seminal plasma contains many morphologically heterogeneous extracellular vesicles (sEVs). These are sequentially released by cells of the testis, epididymis, and accessory sex glands and involved in male and female reproductive processes. This study aimed to define in depth sEV subsets isolated by ultrafiltration and size exclusion chromatography, decode their proteomic profiles using liquid chromatography–tandem mass spectrometry, and quantify identified proteins using sequential window acquisition of all theoretical mass spectra. The sEV subsets were defined as large (L-EVs) or small (S-EVs) by their protein concentration, morphology, size distribution, and EV-specific protein markers and purity. Liquid chromatography–tandem mass spectrometry identified a total of 1034 proteins, 737 of them quantified by SWATH in S-EVs, L-EVs, and non-EVs-enriched samples (18–20 size exclusion chromatography–eluted fractions). The differential expression analysis revealed 197 differentially abundant proteins between both EV subsets, S-EVs and L-EVs, and 37 and 199 between S-EVs and L-EVs *versus* non-EVs-enriched samples, respectively. The gene ontology enrichment analysis of differentially abundant proteins suggested, based on the type of protein detected, that S-EVs could be mainly released through an apocrine blebbing pathway and be involved in modulating the immune environment of the female reproductive tract as well as during sperm–oocyte interaction. In contrast, L-EVs could be released by fusion of multivesicular bodies with the plasma membrane becoming involved in sperm physiological processes, such as capacitation and avoidance of oxidative stress. In conclusion, this study provides a procedure capable of isolating subsets of EVs from pig seminal plasma with a high degree of purity and shows differences in the proteomic profile between EV subsets, indicating different sources and biological functions for the sEVs.

## Highlights

• Two EV subsets (L/S) were isolated from pig seminal plasma by a SEC procedure.
• A total of 1,034 proteins were identified by LC-MS/MS in seminal EVs.
• Up to 737 proteins were quantified by SWATH in seminal EVs.
• The two subsets of seminal EVs showed differences in proteomic profile.
• Each EV subset would have different biogenesis and biological function.

## Graphical Abstract

**Figure:**
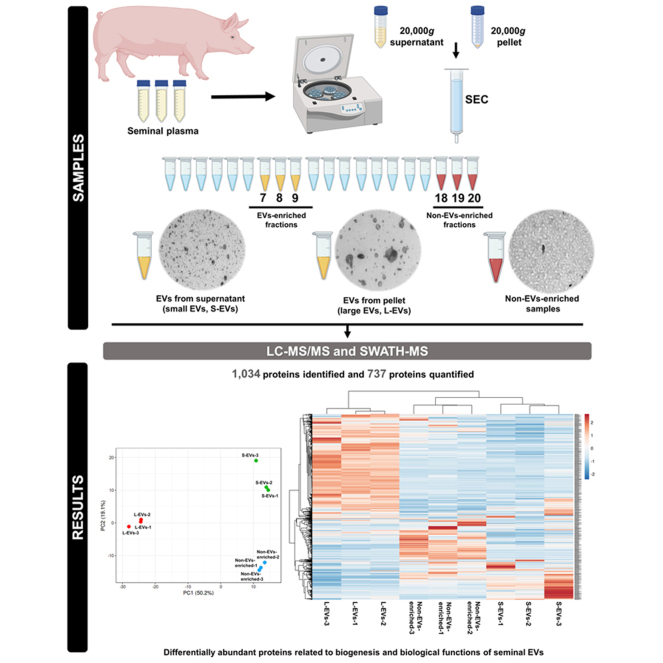

## In Brief

Pig seminal plasma contains a heterogeneous population of extracellular vesicles (sEVs), involved in several reproductive processes. Two sEV subsets (small or large EVs) were isolated by size exclusion chromatography (SEC) followed by a large-scale high-throughput proteomic analysis. A total of 1,034 proteins were identified by LC-MS/MS as encoded into *Sus scrofa* taxonomy, and 737 of them were quantified by SWATH revealing quantitative proteomic differences that suggest different biogenesis and biological functions for the sEVs.

Mounting evidence shows that seminal plasma (SP), the heterogeneous fluid that surrounds sperm during/after ejaculation, plays a key role in many physiological reproductive processes, including sperm function and embryo development (1). This fluid, mainly composed by secretions from the epididymis and accessory sex glands, contains a wide repertoire of biomolecules, as inorganic ions, hormones, lipids, nucleic acids, peptides, and proteins (1). The last of these largely define SP function, as the modulation of sperm function, motility, capacitation, and encounter with the oocyte, as well as triggering immune responses by the female after mating or insemination, is crucial for healthy embryo development (2). Recent research reported that some of these SP proteins could be loaded into seminal extracellular vesicles (sEVs), where they might remain safe from the degradation by proteolytic enzymes in semen (3, 4, 5, 6).

Emerging as a potent mechanism for intercellular communication in the body, EVs are defined as a heterogeneous population of lipid bilayer–enclosed nanovesicles released by virtually all prokaryotes and eukaryotic cells into the extracellular space (7). EVs act as essential messengers in pathological and physiological processes (8, 9), including those involved in reproduction (10, 11, 12, 13, 14). The relevance of EVs lies in their ability to transfer their biologically active cargo (which include a collection of nucleic acids [DNA, mRNA, microRNA], lipids, metabolites and, mainly proteins) to recipient cells, triggering a specific response in them (15). This cargo is variable and highly dependent on EV-secreting cells and biogenesis mechanism (16).

Based on differences in size and biogenesis, the EVs released by healthy cells are classically categorized into exosomes (small EVs of ∼40–150 nm in diameter; endosomal origin) or microvesicles (large EVs of ∼100–1000 nm in diameter; plasma membrane origin) (8). The lack of standardized isolation procedures capable of accurately separating EV subsets has led to a suboptimal definition of their composition and function. Recent data indicate that EV subsets exhibit a different proteomic profile (8, 17, 18), suggesting that they might have distinct biological functions.

At present, sEVs despite being those first identified (19), and being in greater amount than in other biofluids (20), remain poorly explored (21). Research on seminal EVs accounts for less than 1 % of the total studies (22), and therefore, research on sEVs remains a challenge. Currently existing research evidenced that sEVs could be key modulators of sperm functions, including epididymal maturation, motility, capacitation, and acrosome reaction, and ultimately might influence fertilization (23, 24, 25, 26). In pig, sEVs could be involved in the regulation of sperm–oocyte interaction (27, 28, 29) and in the inflammatory and immune responses in the female genital tract, essential for providing cleansing of pathogens and the development and implantation of healthy embryos (30). However, the underlying mechanisms by which sEVs could modulate these processes remain to be elucidated, considering EV-loaded proteins could be key molecules. In recent years, various -omics approaches have enabled the characterization of sEV loads, thereby substantially improving our understanding of their functional roles (5, 31, 32, 33). In human, the proteomic profile of sEVs has been decoded (3, 4, 6, 31, 34), depicting differences in protein composition between EV subsets (4, 6, 35) and suggesting that each sEV subtype could be involved in a specific function. To date, only one study has evaluated the proteomic profile of sEV subsets in cattle (5). In a previous study in pig SP, we identified putative EV subsets according to tetraspanin expression profile (36), suggesting differences in their cargo and biological function.

The present study aimed to define in depth the proteome profile of pig sEVs to (1) compare sEV-enriched *versus* SP-free proteins; (2) identify putative differences in protein composition between sEV size subsets; and (3) analyze, according to their proteomic profile, the potential functional roles of each sEV subtype. For this, sEV size-subsets were isolated using ultrafiltration and size exclusion chromatography (SEC), the proteomic profiles decoded using liquid chromatography–tandem mass spectrometry (LC-MS/MS), and the identified proteins finally quantified using sequential window acquisition of all theoretical mass spectra (SWATH-MS).

## Experimental Procedures

### Animals and Samples

The procedures that involved animals were performed according to international guidelines on the protection of animals used for scientific purposes (Directive 2010-63-EU) and approved by the Bioethics Committee of Murcia University (research code: CBE: 367/2020). All reagents used, unless stated otherwise, were purchased from Merck.

Ejaculates were collected from mature (10–30 months), healthy, and fertile Pietrain artificial insemination (AI) boars housed in an AI center belonging to AIM Ibérica (Topigs Norsvin Spain SLU). All ejaculates used in the experiment (n = 9; one ejaculate per boar) fulfilled semen quality thresholds (˃ 200 × 10^6^ sperm/ml, ˃70% motile sperm, and ˃75% sperm with normal morphology) for commercial production of pig semen AI doses. Immediately after semen collection, the nine ejaculates were pooled, generating three pools. Each pool was centrifuged twice (1500*g* [Rotofix 32A, Hettich Centrifuge UK] at room temperature [RT] for 10 min) for SP harvesting. Then, the three resulting SP sample pools were microscopically examined (Eclipse E400; Nikon) to confirm the absence of sperm. Subsequently, the pooled SP samples were stored at 5 °C (Zanussi Tropic System, Electrolux España S.A.U) in the presence of a protease inhibitor cocktail Roche complete (Protease Inhibitor Cocktail tablets; Basilea) until EVs isolation.

### Extracellular Vesicles Isolation

**Fig. 1:**
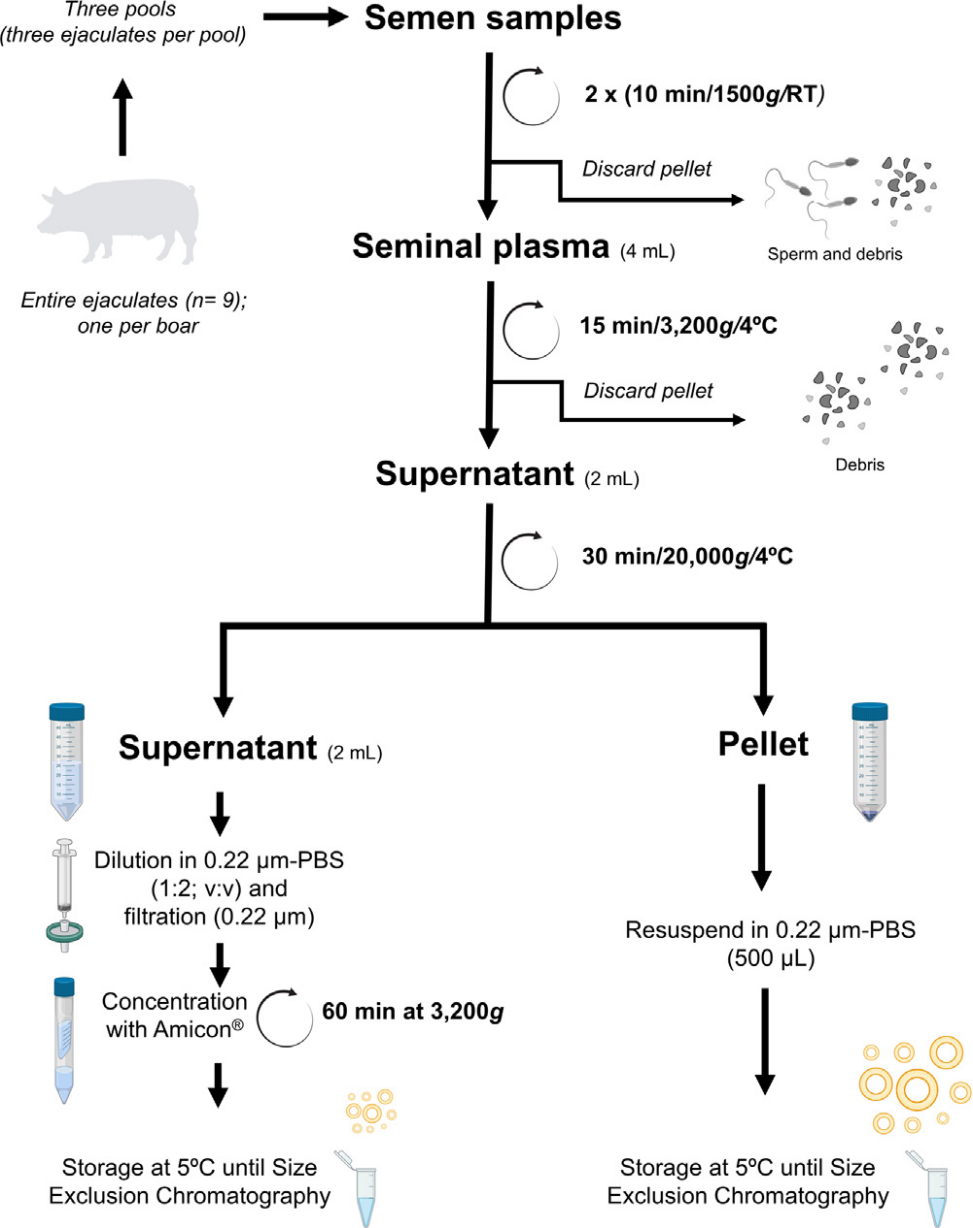
**Flow chart showing the handling of boar seminal plasma prior to isolation of extracellular vesicle subsets**. RT, room temperature. The drawing was created using the software of BioRender.com.

A flow chart of SP processing before ultrafiltration and SEC for EVs isolation is shown in Figure 1. First, the three SP samples (of 4 ml each) were centrifuged (3200*g* at 4 °C for 15 min; Sorvall STR40, Thermo Fisher Scientific) to remove any debris. The collected supernatants (2 ml) were centrifuged (20,000*g* at 4 °C for 30 min; Sorvall Legend Micro 21R, Thermo Fisher Scientific), and the resulting pellets and supernatants were processed in different ways. The pellets were resuspended in 0.22-μm-filtered phosphate buffered saline (PBS), 500 μl, and stored at 5 °C until SEC. The supernatants were diluted in 0.22-μm-filtered PBS (1:2; v:v), filtered (0.22 μm, Millex Syringe Filters), and concentrated (Amicon Ultra 4 ml centrifuge filter 3 kDa) by repeated centrifugations (3200*g* at 4 °C for 90 min). The resultant samples (ranging from 1.5 to 2 ml) were stored at 5 °C until SEC.

**Fig. 2:**
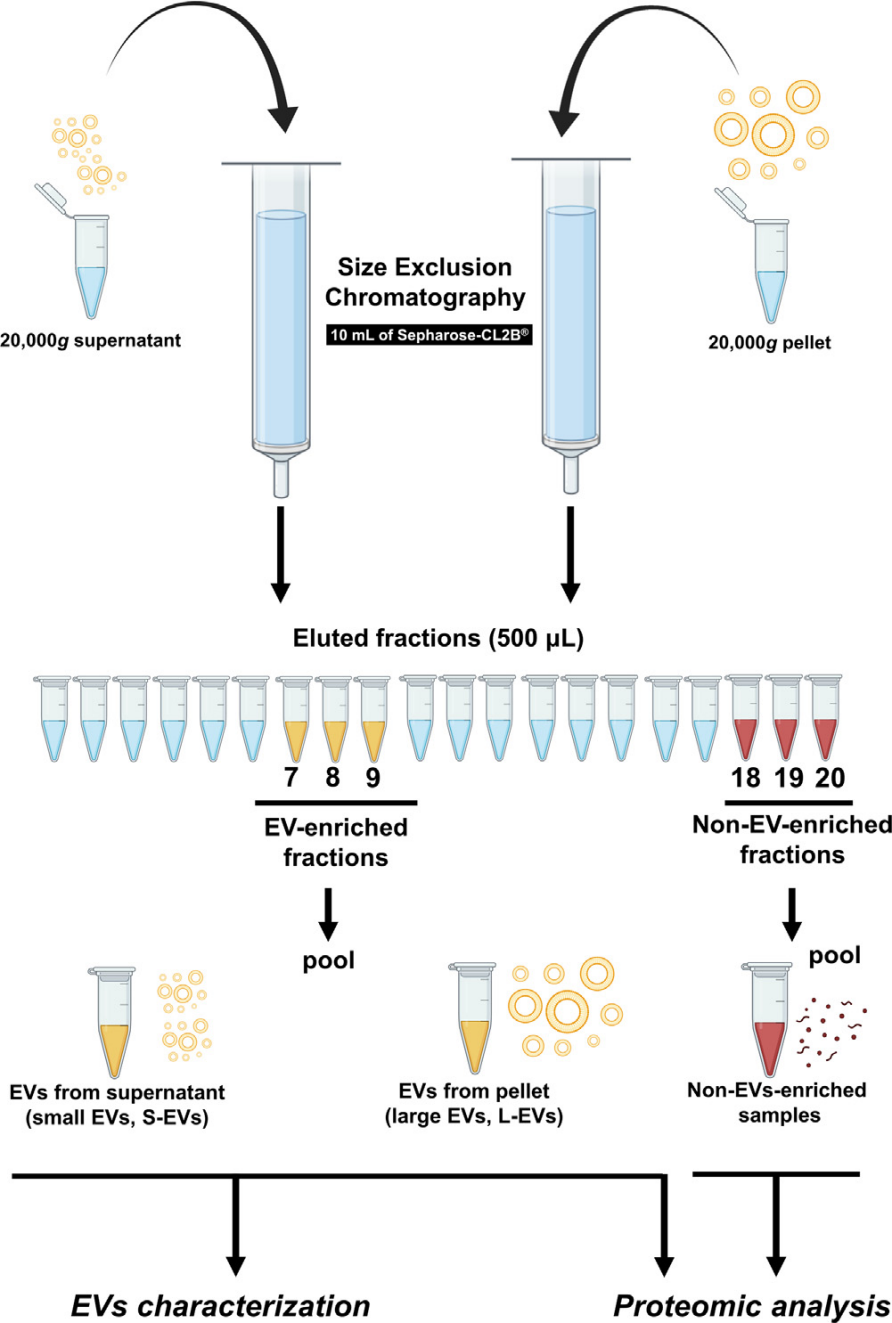
**Schematic summary of the size exclusion chromatography procedure to isolate the two subsets of extracellular vesicles (EVs) from boar seminal plasma and the experimental workflow used for the analysis of EVs**. The resulting EVs were characterized for total protein concentration, morphology, size, presence of specific protein markers, and purity. The drawing was created using the software of BioRender.com

A flow chart of the seminal EVs isolation by SEC and further characterization of isolated EVs is shown in Figure 2. The obtained pellets and supernatants were separately subjected to SEC. Columns were homemade using 12-ml filtration tubes stacked with 10 ml of Sepharose-CL2B. Before EVs isolation, columns were equilibrated by washing them with 0.22-μm-filtered PBS (60 ml). Then, pellet and supernatant samples were loaded on SEC columns followed by elution with 0.22-μm-filtered PBS. A total of 20 sequential 500 μl eluted fractions were separately collected from each SEC-processed sample, and fractions 7 to 9 (enriched in EVs) and 18 to 20 (non-EVs-enriched) were selected. These fractions were separately mixed to generate two samples per SEC procedure. The six non-EVs-enriched samples (three from pellet SECs and three from supernatant SECs) were pooled. In total, nine samples were then generated after SECs isolation; EV samples resulting from either pellets (n = 3; mainly enriched in large EVs [L-EVs]) or supernatants (n = 3; mainly enriched in small EVs [S-EVs]) and the non-EVs-enriched and protein-enriched ones (n = 3; non-EVs-enriched), all of which were stored at −80 °C (Ultra Low Freezer; Haier Inc) until EV characterization and proteomic analysis.

### Characterization of the Extracellular Vesicles

The isolated sEV subsets (S- and L-EVs) were characterized using multiple and complementary approaches (37), following the minimal information for studies of extracellular vesicles 2018 (MISEV 2018) guidelines. Specifically, they were characterized in terms of (1) concentration and size distribution, by assessing total protein concentration and using dynamic light scattering (DLS) analysis and nanoparticle tracking analysis (NTA); (2) morphology, using transmission electron microscopy (TEM); (3) specific protein markers (CD63, HSP90β); and (4) EV purity (albumin content), the last two characterizations using flow cytometry. Details of this EV characterization are given below. The non-EVs-enriched samples were also analyzed using the same procedures to check whether they contained EVs or not.

#### Determination of Total Protein

The concentration of total protein was measured using the Bicinchoninic Acid Assay following manufacturer instructions (Thermo Scientific). Prior to the analysis, EV samples were lysed. For this purpose, 25 μl of EV samples was mixed with 25 μl of lysis solution (0.1% of Triton plus 0.1% of sodium dodecyl sulphate [SDS]) and incubated at 37 °C for 30 min under shaking (50 rpm). Absorbance was determined using a microplate reader (PowerWave XS; Bio-Tek Instruments) at a wavelength of 570 nm.

#### EV Concentration and Size Distribution

The concentration and size distribution of EVs (S-EVs and L-EVs) were assessed by NTA using a NanoSight LM10 (Malvern Instrument Ltd), equipped with a 405-nm laser and a scientific complementary metal oxide–semiconductor camera. The recorded data were analyzed with the NTA software (version 3.3.; Dev Build 3.3.104), with Min track Length, Max Jump Distance, and Blur set to auto and the detection threshold set to five. The camera level was established to 15, and 5 videos of 30-s duration at 30 frames/s were captured. The images were recorded with manual monitoring of temperature. Following the manufacturer recommendations, the number of particles in the field of view of the EV samples was reduced to 20 to 120 particles/frame with 0.22-μm-filtered PBS.

The hydrodynamic size and size distribution of EVs were measured by DLS using a Zetasizer Nano ZS-system (Malvern Panalytical) operating at 633 nm at RT, recording the back scattered light at an angle of 173°. Briefly, 50 μl of each EV sample was shacked to avoid potential EV clumps and loaded into a cuvette with a 10-mm path length. The light scattering was recorded for 150 s, with three measurements carried out per sample. The conversion of DLS signal intensity to size distribution was performed using a Dispersion Technology Software v.5.10 (Malvern Panalytical). The EV diameter (in nm) was calculated using the peak maximum of the Gaussian function. The intensity-based distribution was recalculated to the volume, and the results were expressed as intensity- and volume-size distribution.

#### EV Morphology

The morphology of EVs (S-EVs and L-EVs) was visualized by TEM following the protocol described by Thery *et al*. (38) with minor modifications. Briefly, 10 μl samples were fixed in 2% paraformaldehyde for 30 min and placed onto carbon-coated copper grids for 15 min. After washing with 0.22-μm-filtered PBS, the EV samples were fixed for 5 min with 1% glutaraldehyde. Then, the samples were washed with distilled water, contrasted in 1% uranyl acetate, and soaked in 0.5% methylcellulose. Finally, the samples were dried at RT before visualization using a JEOL JEM 1011 transmission electron microscope (JEOL Ltd) at 80 kV at the Servicio Central de Soporte a la Investigacion Experimental, Universitat de València. The EV diameter was assessed by ImageJ 1.41 software (National Institutes of Health).

#### Flow Cytometry

The analyses were performed with the high-resolution flow cytometer CytoFLEX S (Beckman Coulter, Life Sciences Division Headquarters), equipped with violet (405 nm), blue (488 nm), yellow (561 nm), and red (638 nm) lasers. Recombinant EVs expressing green fluorescent protein on their membrane surface (SAE0193, Merck) were used to verify the accuracy of the flow cytometer for EV input and counting. The optical setup was adjusted to use the side scatter (SSC) information from the 405-nm laser (Violet-SSC-A). The forward scatter (FSC) and Violet-SSC-A were adjusted on a logarithmic scale, and the fluorescence channels adjusted on a logarithmic gain. The analysis was restricted to events with size (FSC) and complexity (Violet-SSC-A) characteristics specific to EVs. Samples were analyzed using the low flow setting (10 μl/min) acquiring at least 10 × 10^3^ events per sample. Distilled water (filtered 0.1 μm) was used as sheath fluid, while 0.1-μm-filtered PBS ensured removal of background noise.

The EVs were cytometrically characterized following the International Society of Extracellular Vesicles recommendations (MIFlowCyt-EV, (39)) to identify their enrichment in proteins belonging to the three categories established by MISEV 2018 guidelines (37): CD63 (Anti-CD63-FITC, Clone REA1055, Miltenyi Biotec) as “Category 1” protein (Transmembrane or GPI-anchored proteins associated to plasma membrane and/or endosomes); HSP90β (anti-HSP90β-PE, ADI-SPA-844PE-050, Enzo Life Sciences) as “Category 2” protein (Cytosolic proteins recovered in EVs), and albumin (Anti-swine Albumin-FITC, CLFAG16140, Cedarlane) as “Category 3” protein (Major components of non-EVs coisolated structures). First, 10 μl of each EV sample was incubated with CellTrace CFSE to discriminate intact EVs from membrane fragments. Thereafter, each EV sample was split into three aliquots, which were incubated with anti-HSP90β-PE, anti-CD63-FITC, or anti-Albumin-FITC at RT for 30 min. Then, samples were diluted in 0.1-μm-filtered PBS to a final volume of 300 μl and analyzed in CytoFLEX S. CFSE-labeling was used to backgated events in the EV gate (Violet SSC-H and FSC-H).

### Proteomic Analyses

Proteomics was performed in the Proteomics Unit of the SCSIE, Universitat de València (PRB2-ISCIII ProteoRed Proteomics Platform member). A portion of the individual samples was mixed to generate three pools (one per type of sample: L-EVs, S-EVs, and non-EVs-enriched). Individual samples (L-EVs and S-EVs) and pooled samples (L-EVs, S-EVs, and non-EVs-enriched) were used to decipher the proteomic profile (for spectral library generation), and individual samples (L-EVs, S-EVs, and non-EVs-enriched) were used to quantify the identified proteins to distinguish differentially expressed ones among samples.

#### Protein Extraction

Total protein concentration of each sample was measured using a Qubit fluorometer (Invitrogen) following manufacturer instructions.

#### SDS-PAGE and In-Gel Digestion

Forty micrograms (for S-EVs and non-EVs-enriched) and 20 μg (for L-EVs) of total protein were mixed (4:1; v:v) with 4 × Laemmli sample buffer (Bio-Rad) containing β-mercaptoethanol and denatured at 95 °C for 5 min. Then, samples were loaded on 12% Tris HCl precast one-dimensional SDS-PAGE (Bio-Rad) including a molecular weight marker (ECL Plex Fluorescent Rainbow Marker, GE Healthcare Life Sciences). The electrophoresis was performed at a constant voltage of 200 V for 5 min at RT. Then, the gel was fixed with a solution containing 40% ethanol and 10% acetic acid for 60 min, stained with Brilliant R250 Blue stain Coomassie (Bio-Rad) for 60 min to visualize protein bands after destaining with H_2_O milliQ. Thereafter, for pooled samples the lanes were sliced into six pieces (non-EVs-enriched) and five pieces (L-EVs and S-EVs), respectively. For individual samples, the gel was sliced at 25 kDa into two pieces (for S-EVs and non-EVs-enriched), and the top and the bottom pieces of the gel were used to analyze the less and major abundant proteins, respectively. Finally, the gel from L-EVs was cut into a single slice. All samples were digested following the protocol used by Shevchenko *et al*. (40). Briefly, this procedure involves (i) trypsin digestion (ranging from 150 to 500 ng; Promega) at 37 °C, stopped with 10 % trifluoroacetic acid; (ii) peptide extraction by incubation in pure acetonitrile at 37 °C in a shaker for 15 min; and (iii) suspension of the resulting peptide mixture in 2% acetonitrile and 0.1% trifluoroacetic acid (6–30 μl), after drying in a speed vacuum (ISS 110 SpeedVac System, Thermo Savant, Thermo Scientific).

#### LC-MS/MS Analysis

The peptide mixtures were analyzed for the spectral library acquisition by liquid chromatography (LC) using a NanoLC Ultra 1D plus (Eksigent Technologies) connected to an AB SCIEX TripleTOF 6600^+^ mass spectrometer (AB SCIEX) in direct injection mode. Briefly, 5 μl of peptide mixture (obtained from in-gel digestion of each sample) was loaded on a trap NanoLC precolumn (3 μm particle size C18-CL, 350 μm × 0.5 mm; Eksigent Technologies) and desalted with 0.1% trifluoroacetic acid at 5 μl/min for 5 min. Thereafter, the digested peptides were loaded onto an analytical LC column (3 μm particle size C18-CL 120 A, 0.075 × 150 mm, Eksigent Technologies) equilibrated in 5% acetonitrile and 0.1% formic acid (Thermo Fisher Scientific). Then, peptide elution was carried out using a linear gradient from 7% to 40% of acetonitrile containing 0.1% formic acid at a constant flow rate of 300 nl/min for either 45 min (pooled samples and less abundant proteins of individual samples [L-EVs and S-EVs]) or 20 min (major abundant proteins of individual samples [L-EVs and S-EVs]), respectively. The eluted peptides were infused on a spectrometer nanoESI qQTOF (TripleTOF 6600^+^). The samples were ionized using an Optiflow system applying 3.0 kV to the emitter spray at 200 °C. The TripleTOF was operated in data-dependent mode, in which a time of flight (TOF) mass spectrometry (MS) scan was carried out from 350 to 1400 *m/z* and accumulated for 250 ms. The quadrupole resolution was set to “LOW” for MS2 experiments, which were acquired from 100 to 1500 *m/z* for 25 ms in “high sensitivity” mode. The criteria for precursor peptide ions selection were charge (2+, 3+, or 4+) and minimum intensity of 250 counts per second. Ions with 1+ and unassigned charge states were removed from the analysis. Up to 100 ions were selected for fragmentation after each scanning, and dynamic exclusion was set to 15 s. The rolling collision energy equations were automatically set by the instrument.

#### LC-SWATH-MS Acquisition

To determine quantitative differences between the L-EVs *versus* S-EVs, S-EVs *versus* non-EVs-enriched, and L-EVs *versus* non-EVs-enriched samples, each individual sample was analyzed configuring the TripleTOF 6600^+^ as described by Gillet *et al*. (41) for SWATH-MS-based experiments. Briefly, 5 μl (less abundant proteins) and 1 μl (major abundant proteins) of each sample were randomly loaded onto a trap column (3 μm particle size C18-CL, 350 μm × 0.5 mm; Eksigent Technologies) and desalted with 0.1% trifluoroacetic at 5 μl/min for 5 min. Then, peptides were loaded onto an analytical column (3 μm particle size C18-CL 120 A, 0.075 × 150 mm, Eksigent Technologies) equilibrated in 5% acetonitrile and 0.1% formic acid. Then, peptide elution was carried out using a linear gradient from 5% to 40% of acetonitrile containing 0.1% formic acid at a constant flow rate of 300 nl/min for 20 min for major abundant proteins or for 45 min for less abundant proteins. The analysis of eluted peptides was carried out in a mass spectrometer nanoESI qQTOF (TripleTOF 6600^+^). The TripleTOF operated in SWATH mode (data-independent mode), in which a 0.050-s TOF MS scan from 350 to 1250 *m/z* was performed, followed by 0.020-s product ion scans from 350 to 1250 *m/z* split into 100 widths windows from 400 to 1250 Da (3.05 s/cycle). The collision energy for each window was calculated for 2+ charged ion at the center of each SWATH block with a collision energy spread of 15 eV.

#### Spectral Library Generation and Protein Quantitation

The .wiff data files obtained were processed by Protein Pilot v5.0 search engine (AB SCIEX). The Paragon algorithm (4.0.0.0, 4767) of ProteinPilot v5.0 was used to search against UniProt Database (UniProt-Mammalia_200218.fasta) with the specific parameters: trypsin specificity, cys-alkylation, no taxonomy restriction (120,117 proteins in the database and 240,234 interrogated proteins [sum of target and decoy proteins for false discovery rate (FDR) calculation]), and the search effort set to through. The same analysis was also made for *Sus scrofa* taxonomy. Irrespective of the peptide sequence assigned, and with the aim of avoiding using the same spectral evidence in more than one protein, the identified proteins were grouped based on tandem mass spectrometry (MS/MS) spectra by the Protein-Pilot Pro Group Algorithm. The primary protein of the group was defined as the protein within each group that could explain the most spectral data with confidence. An FDR, calculated by Protein-Pilot Pro Group Algorithm, threshold of ≤1% was established.

The .wiff files obtained were analyzed by PeakView (v2.1, AB SCIEX) by MS/MS^ALL^ with SWATH Acquisition MicroApp 2.0.1(https://download.sciex.com/SWATH_Processing_Release_Notes.pdf?_ga=1.107240321.2033308351.1472479602), and peaks from SWATH were extracted with a peptide confidence threshold ≥95%. An FDR threshold of ≤1%, 100 peptides per proteins, 6 transitions per peptide, excluding modified peptides, were established for analysis. In the same way, extracted ion chromatogram (XIC) extraction window (min) was established in 7 and XIC width (ppm) in 50. The XIC of every peptide was integrated, and the peak areas were used to calculate total protein. The protein areas were normalized among samples by total sum. For each protein, the identifier and gene name were extracted from the UniProt Database.

#### Bioinformatics

Gene Ontology (GO) enrichment (including cellular component, molecular function, and biological process) and Kyoto Encyclopedia of Genes and Genomes pathway analysis of differentially abundant proteins was performed using the online bioinformatics tools UniProt KB (https://www.uniprot.org/) and Database for Annotation, Visualization and Integrated Discovery (DAVID; https://david.ncifcrf.gov/). Analyses were conducted in Partek Genomics Suite software (version XCC, Partek Inc) to determine the top enriched GO terms for each sample type. The major categories (cellular component, molecular function, and biological process) were broken down into second-level GO terms. Figures were generated using GraphPad Prism 9.3.0 (GraphPad Software, Inc).

### Experimental Design and Statistical Rationale

Nine ejaculates (one ejaculate per mature and fertile boar) were mixed in three pools to diminish individual effects (three ejaculates per pool), which were centrifuged for SP harvesting (as described above). The three resulting SP samples were subjected to SEC-based procedure for EV subsets isolation (as described above). Nine samples were obtained: (1) enriched in L-EVs, n = 3; (2) enriched in small EVs, n = 3; and (3) non-EVs-enriched and protein-enriched ones, n = 3. The isolated EVs were characterized following MISEV 2018 guidelines (as described above). Qualitative and quantitative proteomic analyses were carried out in the nine samples following the procedure described above. Specifically, individual samples (L-EVs and S-EVs) and pooled samples (L-EVs, S-EVs, and non-EVs-enriched) were used for spectral library generation (LC-MS/MS), and individual samples (L-EVs, S-EVs, and non-EVs-enriched) were used to quantify the identified proteins to identify differentially expressed ones among samples (SWATH-MS).

Protein data were statistically analyzed using the R statistical package (https://www.r-project.org). The Shapiro–Wilk test was used to test for normality, and one-way ANOVA was performed to identify differentially abundant proteins among samples (S-EV, L-EV, and non-EVs-enriched). Principal component analysis and heatmap of global protein profiles were carried out by ClustVis (https://bio.tools/clustvis) (42). A fold change >2 or <−2 with *p*-value <0.05 was used to identify the differentially abundant proteins between samples.

The statistical package Prism 9.3.0 was used to analyze data related to the characterization of EV subsets. The normal distribution of the data was assessed by the Shapiro–Wilk test. The data were then analyzed by one-way ANOVA test, and pairwise differences between samples (L-EV, S-EV, and non-EVs-enriched) were confirmed by Tukey’s multiple comparison test. Differences were considered statistically significant at *p* < 0.05.

## Results

### Characterization of the EVs Isolated from Pig Seminal Plasma

**Fig. 3:**
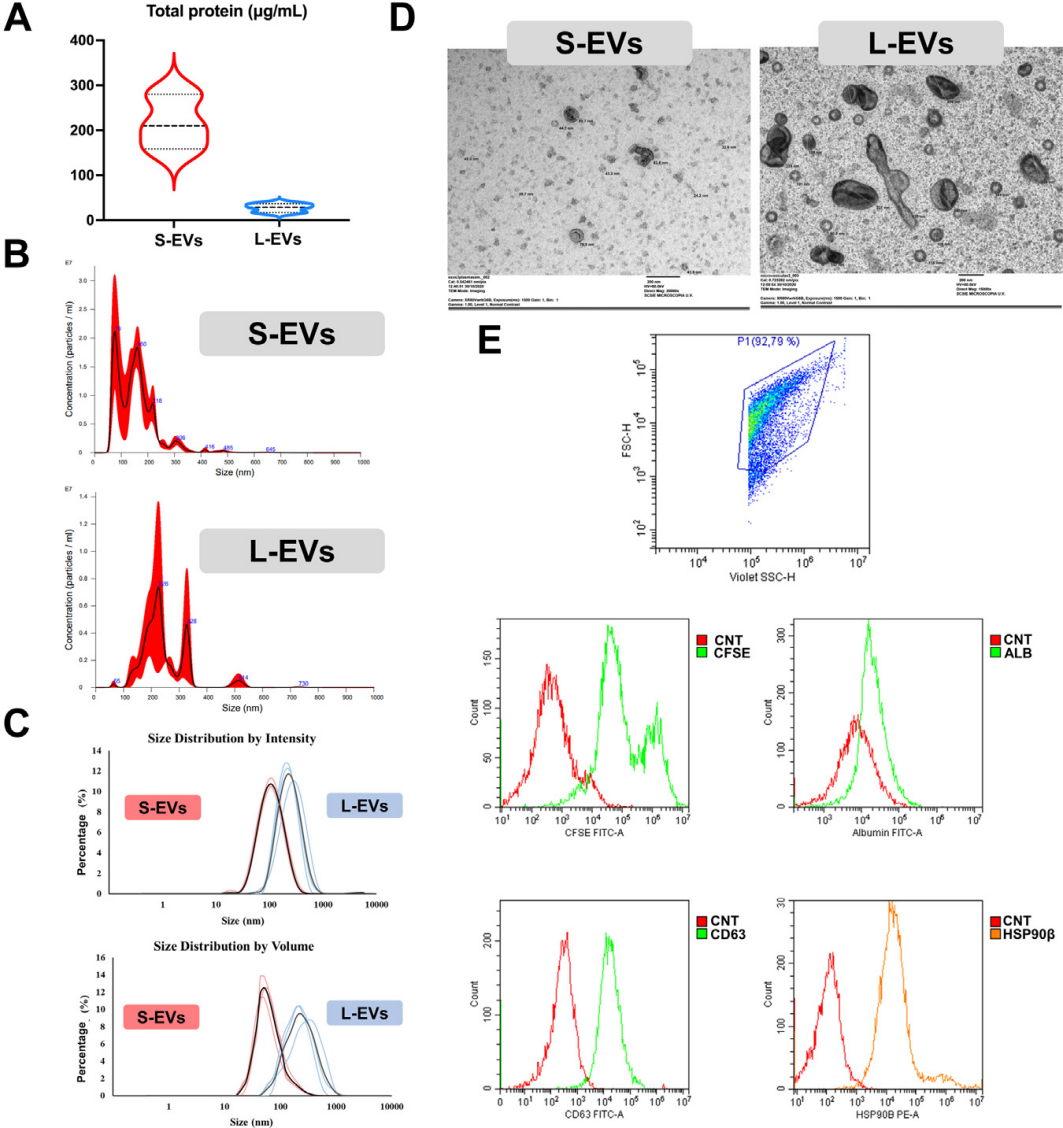
**Characterization of extracellular vesicle (EVs) subsets, namely, small (S-EVs) or large (L-EVs), isolated from pig seminal plasma (SP) samples (n = 3; three ejaculates per sample; one ejaculate per male pig).***A*, violin plot displaying the total protein concentration in both SP-EV subsets. The *dashed line* shows the median and dotted lines the 25 to 75% quartiles. *B*, representative histogram of particle size distribution of S-EVs and L-EVs assessed by nanoparticle tracking analysis. *C*, particle size distribution of S-EVs and L-EVs analyzed by dynamic light scattering (*red*, S-EVs; *blue*, L-EVs) in terms of intensity and volume. The *black* and *gray lines* represent the average of intensity size distribution of S-EVs and L-EVs, respectively. *D*, representative images of the morphology of S-EVs and L-EVs assessed by transmission electron microscopy. *E*, representative histogram of CFSE/CD63/HSP90β/ALB expression in S-EVs and L-EVs assessed by flow cytometry. ALB, albumin; CFSE, carboxyfluorescein succinimidyl ester; CNT, control; HSP90β, heat shock protein 90β.

Total protein concentration (mean ± SD) was higher (*p* < 0.001) in S-EV (216 ± 61 μg/ml) than in L-EV samples (24.2 ± 15.3 μg/ml) from pig SP (Fig. 3*A*). NTA revealed that the particle concentration (mean ± SD) was higher (*p* < 0.01) in S-EV samples (13.6 × 10^11^ ± 3.65 × 10^11^ particles/ml) than in L-EV samples (3.7 × 10^11^ ± 9.06 × 10^10^ particles/ml). As expected, the NTA showed that EVs were smaller (*p* < 0.05) in S-EV samples (diameter, mode ± SD: 151.47 ± 19.87 nm;) than in L-EVs samples (diameter: 196.87 ± 21.60 nm) (Fig. 3*B*). DLS analysis confirmed that EVs were smaller (*p* < 0.01) in S-EV samples (average diameter of ∼100–125 nm) than in L-EV samples (average diameter of ∼230–290 nm) (Fig. 3*C*).

Imaging by TEM showed the S-EV samples contained spherical membranous vesicles ranging in size from 30 to 100 nm, consistent with the size described for exosomes. The L-EV samples contained, however, a morphologically heterogeneous population of membranous vesicles ranging in size from 100 to 350 nm, consistent with the size that characterizes microvesicles (Fig. 3*D*). The TEM images also showed both L-EV and S-EV samples contained subsets of EVs with clear electron density differences. Apoptotic bodies, debris, or protein aggregates were absent in either subset sample (Fig. 3*D*). Although size differences were clear between S- and L-EV samples, TEM images revealed the presence of small EVs in the L-EV samples and some large EVs in the S-EV samples (Fig. 3*D*).

**Supplementary Figure S1:**
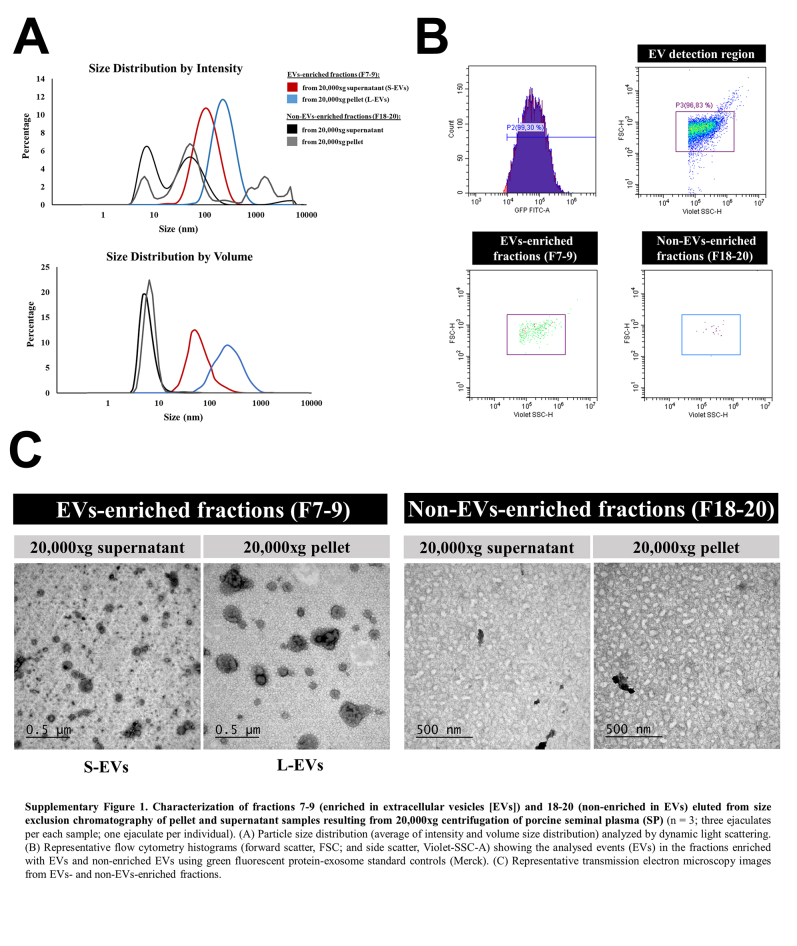


Flow cytometry analysis revealed that both EV subsets contained typical EV markers such as CD63 (mean ± SD, 77.62 ± 6.89% and 78.51 ± 6.57% for S-EV and L-EVs, respectively) or HSP90β (90.74 ± 4.02% and 78.06 ± 14.82%). A high-purity EV enrichment was also confirmed in both EV subsets, as the percentage of albumin was very low (2.84 ± 1.38 and 3.28 ± 1.07 for S-EV and L-EV, respectively) (Fig. 3*E*). DLS, TEM, and flow cytometry analyses confirmed that fractions 18 to 20 eluted from SEC, the so-called non-EV-enriched samples, and contained very few EV-like sized particles (supplemental Fig. S1).

### Proteomic Analysis

#### Protein Repertory

An in-depth proteomic analysis was carried out in L-EV, S-EV, and free EV samples. The raw LC-MS/MS dataset was generated from three pools (one per each type of sample) and the three individual samples of L-EVs and S-EVs (supplemental Table S1). A total of 120,117 proteins were searched in the database and 240,234 proteins interrogated (sum of target and decoy proteins for FDR calculation). A total of 1,034 proteins with an FDR ≤1% were identified as encoded into *S. scrofa* taxonomy, and 988 of them showed a confidence limit of 95% (supplemental Table S2; annotated spectra may be accessed *via* Data Availability section).

**Supplementary Figure S2:**
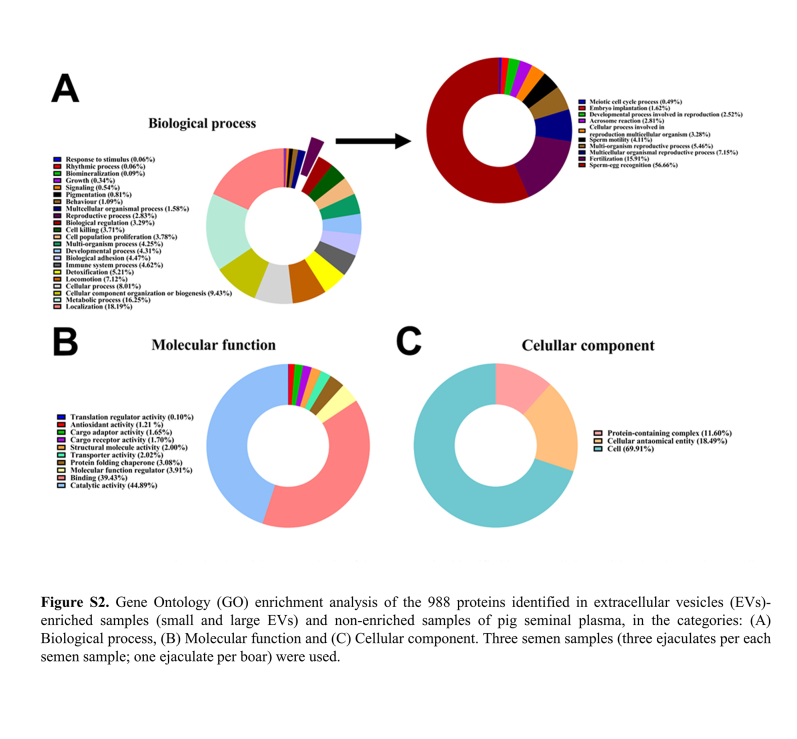


A GO enrichment analysis (supplemental Fig. S2) was then performed. The biological process analysis displayed a noticeable variety of biological roles, most proteins being primarily involved in localization, metabolic processes, and cellular component organization or biogenesis. In relation to reproductive processes, the analysis revealed that most proteins were involved in sperm–egg recognition and fertilization. The analysis also revealed an enrichment of proteins with catalytic and binding activity. Concerning cellular compartment, the proteins belonged to three principal categories, specifically cell, protein-containing complex, and cellular anatomical entity.

#### Quantitative Protein Analysis

**Fig. 4:**
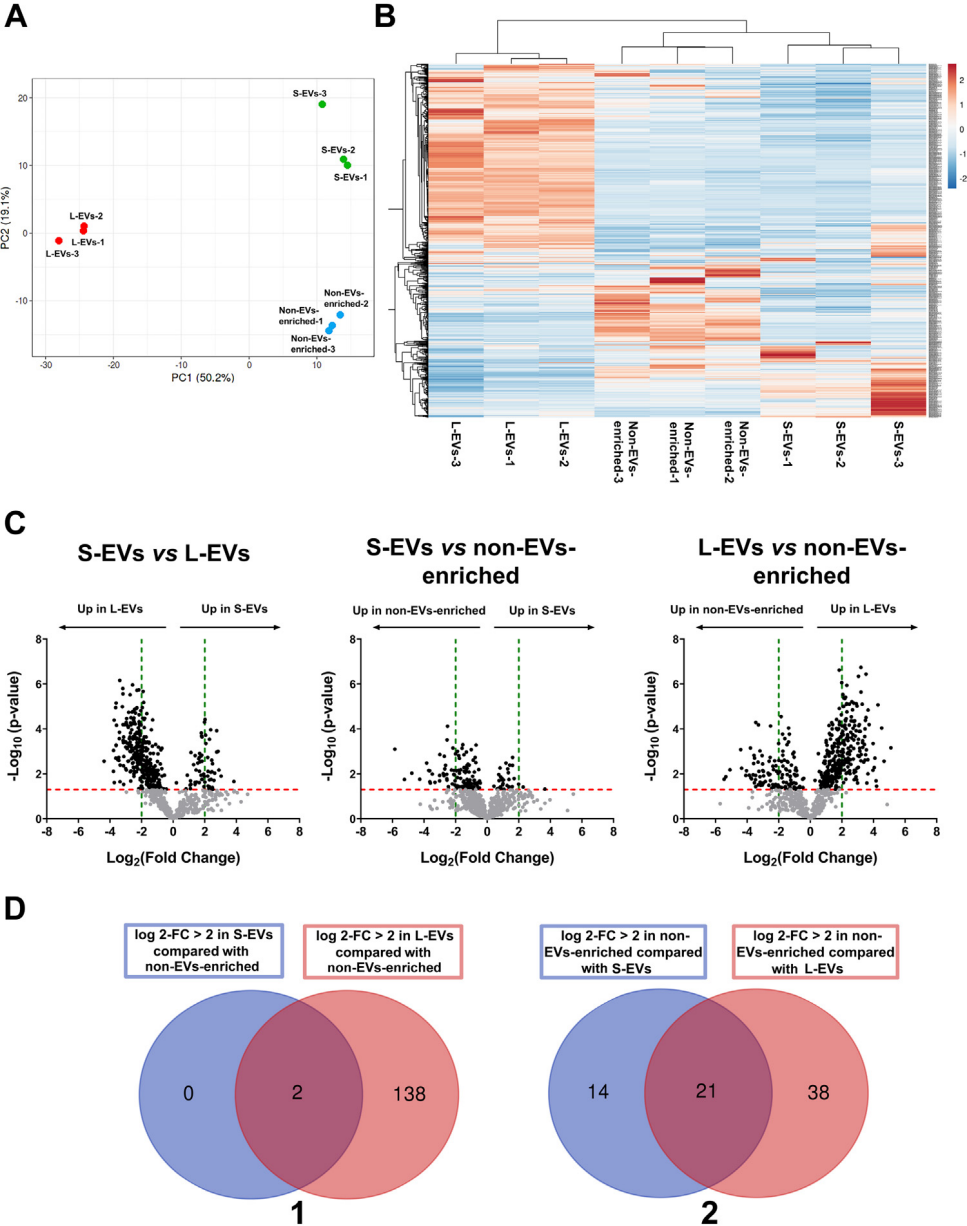
**Proteomic profiling of the two subsets of pig seminal extracellular vesicles (EVs) (small [S-EVs]andlarge [L-EVs]) and ofnon-EV-enrichedsamples (n = 3; three ejaculates per each sample; one ejaculate per individual).***A*, two-dimensional principal component analysis evaluating differences on the quantified proteins in S-EVs, L-EVs, and non-EVs-enriched samples. *B*, heatmap depicting the abundance patterns of 737 proteins quantified in the S-EVs, L-EVs, and non-EVs-enriched samples. Each column and row represents an individual sample and protein, respectively. Relative protein levels are depicted in color scale: *red* indicates more abundance and *blue* indicates less abundance. *C*, volcano plot showing the differentially abundant proteins between the pairwise compared S-EVs, L-EVs, and non-EVs-enriched samples. The *x*-axis shows the log_2_ fold change of the comparation, while the *y*-axis shows the −log_10_ of the calculated probability (*p* value). The dots indicate the proteins that are most abundant in each sample. The *horizontal red line* indicates *p-*value = 0.05, and the *vertical green line* indicates log_2_ fold change = ±2. *D*, Venn diagram showing the number of differentially abundant proteins (with log_2_ fold change > 2; *p*-value ˂ 0.05) between EVs samples (S-EVs or L-EVs) and non-EVs-enriched samples of pig seminal plasma. (1) More abundant in EVs samples and (2) more abundant in non-EVs-enriched samples.

Quantitative proteomics was performed using the SWATH approach. Of the 988 identified proteins, 737 were quantified. All the quantified proteins were found in the three samples, i.e., S-EVs, L-EVs, and non-EVs-enriched. Principal component analysis explained 69.3% of the total variance and clearly discriminated between L-EV, S-EV, and non-EVs-enriched samples (Fig. 4*A*). Specifically, PC1 explained 50.2% and discriminated L-EV samples from S-EV and non-EVs-enriched samples, and PC2 explained 19.1% discriminating between S-EV and non-EVs-enriched samples. Consistent with the results of the PC1, the heatmap of quantified proteins highlighted two main clusters, one with the L-EV samples and the other grouping the S-EV and non-EVs-enriched samples (Fig. 4*B*). This second cluster was in turn divided into two subclusters, one with the S-EV samples and the other with the non-EVs-enriched samples (Fig. 4*B*), which would be consistent with PC2.

A differential expression analysis was performed to identify the proteins that showed the greatest quantitative differences between samples. A log_2_ fold change >2 and *p*-value <0.05 were used as cutoff criteria, and the inventory of the differentially abundant proteins is shown in supplemental Table S4. Volcano plots illustrate the differentially abundant proteins in the pairwise comparisons of the samples (Fig. 4*C*). When EVs (S-EVs and L-EVs) were compared with non-EVs-enriched samples, 35 proteins were less abundant and two more abundant in S-EV, while 59 proteins were less abundant and 140 more abundant in L-EV samples (Fig. 4*C*). Looking at the differentially abundant proteins shared in the EV samples, there were only two more abundant proteins, namely, TMBIM1 and BASP1, against 21 less abundant proteins (Fig. 4*D* and supplemental Table S5). A total of 168 proteins were less abundant and 29 more abundant in S-EV when compared with L-EV samples. Table 1 shows the 20 proteins exhibiting the highest abundant differences between samples.

**Table 1:** The 20 proteins with the largest log_2_ fold change (*p*-value ˂ 0.05) among those differentially abundant between the two subsets of isolated extracellular vesicles (EVs; large [L-EVs] and small [S-EVs]) from pig seminal plasma (n = 3). The proteins most abundant in L-EVs and S-EVs samples are colored in red and green, respectively.

**Fig. 5:**
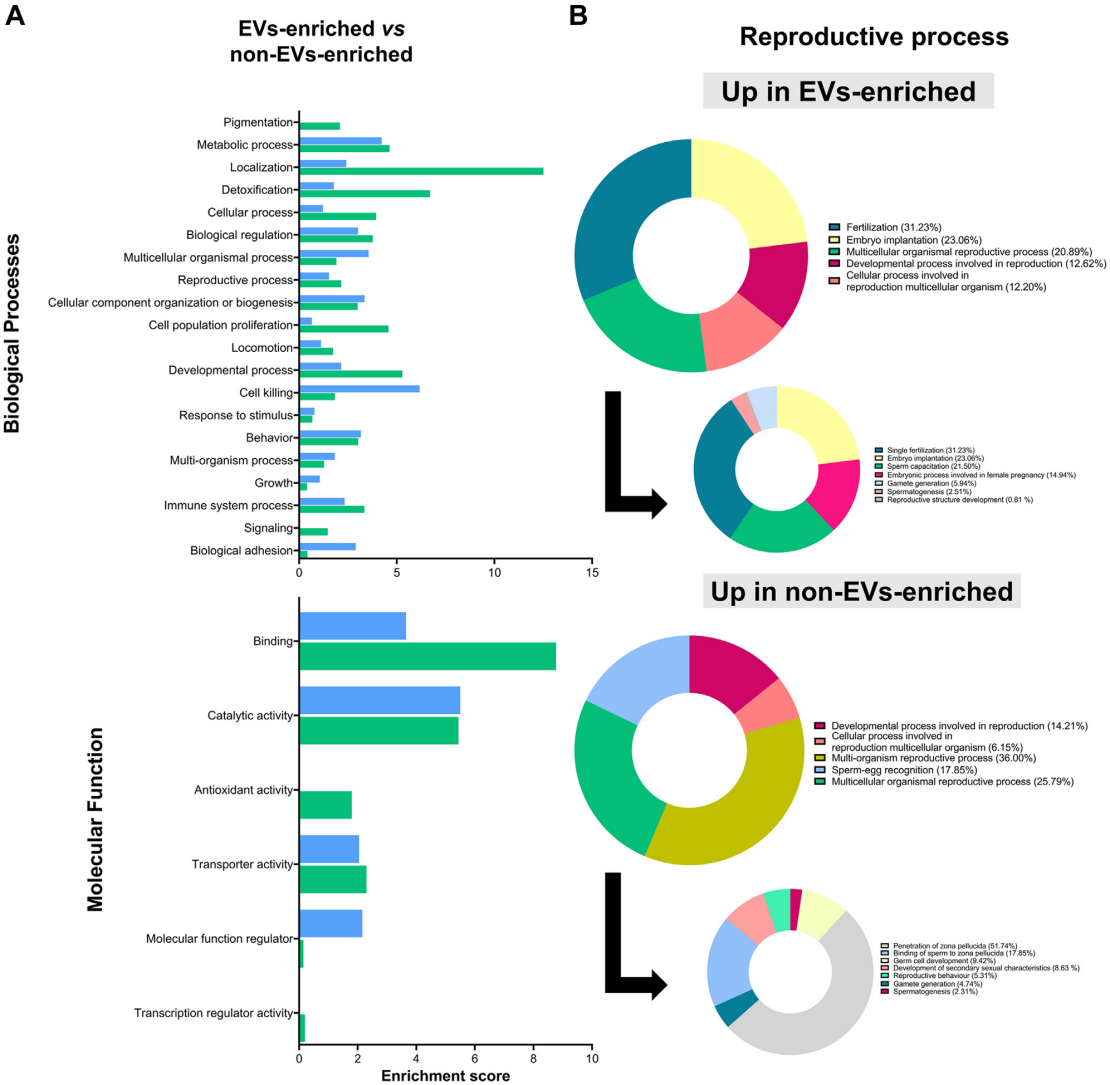
**Gene Ontology enrichment analysis of differential abundant proteins between extracellular vesicles (EVs) samples (small EVs [S-EVs] or large EVs [L-EVs]) andnon-EVs-enrichedsamples of pig seminal plasma.***A*, the terms are ranked by their enrichment scores that were overrepresented in the highly abundant proteins of EVs samples (140 proteins; *green*) or in the non-EVs-enriched samples (73 proteins; *blue*). *B*, reproductive processes in which the enriched are involved.

Functional enrichment analyses of differentially abundant proteins were performed to elucidate possible functional differences between the two subsets of EV (S-EV and L-EV) with circulating free SP proteins (non-EVs-enriched samples). In the first analysis, the most abundant proteins in both EV samples were compared with the most abundant proteins in the non-EVs-enriched samples. The results revealed that the most abundant proteins in the EV samples related to localization, detoxification, and encapsulating development process, while the most abundant proteins in the non-EVs-enriched samples were linked to cell killing, metabolic process, and multicellular organism process (Fig. 5*A*). Regarding reproductive processes, the results revealed that the most abundant proteins in EV samples were involved in fertilization, embryo implantation, and reproductive process in multicellular organisms. The most abundant proteins in non-EVs-enriched samples were involved in reproductive processes in multiorganisms, in multicellular organisms, and in sperm–egg recognition (Fig. 5*B*). In this context, ACSL4, CLIC4, ELSPBP1, GPX4, PEBP1, SLC26A3, and SOD1 were more abundant in EV-samples, and ACE, B4GALT1, and HEXB in those not enriched in EVs. Molecular functional analysis revealed that most of the differentially abundant proteins between EV samples and non-EVs-enriched samples were involved in catalytic and binding activities (Fig. 5*A*). Pathway analysis revealed that most abundant proteins in EV samples were related to gap junction and glycolysis/gluconeogenesis, whereas those most abundant in non-EVs-enriched samples were related to lysosome and glycans degradation (supplemental Table S6).

**Fig. 6:**
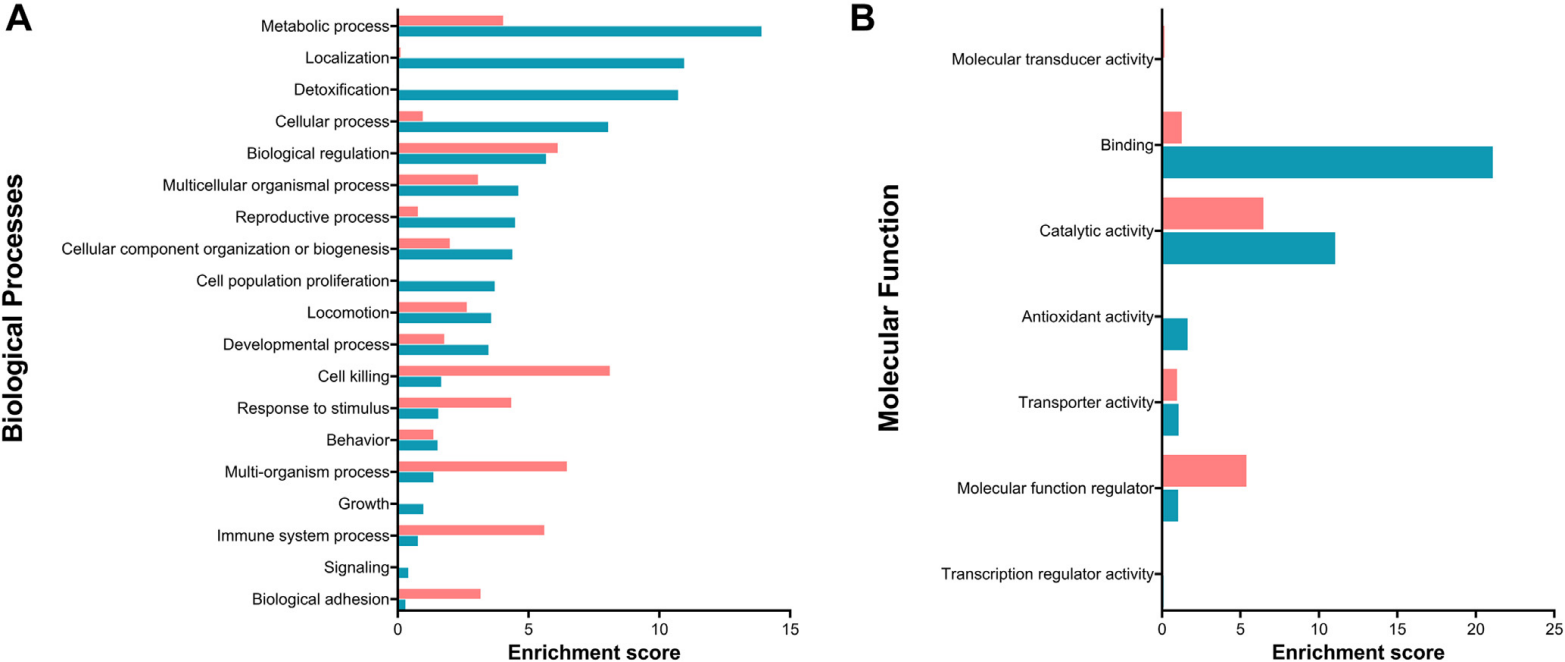
**Gene Ontology enrichment analysis of differentially abundant proteins between the two subsets of extracellular vesicles (EVs) isolated from pig seminal plasma (small [S-EVs] or large [L-EVs]).** Terms are ranked by their enrichment scores, which were overrepresented in the highly abundant proteins of S-EVs (29 proteins; *pink*) or L-EVs (168 proteins; *blue*) samples.

Functional enrichment analysis of differentially abundant proteins between L-EV and S-EV samples was later performed to further elucidate possible functional differences between the two EV subsets. The GO biological process analysis showed most abundant proteins in L-EV samples were involved in metabolic processes, detoxification, and localization, whereas most abundant proteins in S-EV samples were involved in cell killing, immune system process, and multiorgan processes (Fig. 6*A*). Molecular function analysis showed most abundant proteins in L-EV samples had catalytic and binding activity, while those most abundant proteins in S-EVs had catalytic and regulatory activity (Fig. 6*B*). Regarding differentially abundant proteins involved in reproduction-related processes, those most abundant in L-EV samples were SOD1, CLIC4, PAFAH1B2, ELSPBP1, GPX4, PGK2, GRB2, SLC26A3, SRC, and PRKACA, while B4GALT1 prevailed in S-EV samples. The pathway analysis linked most abundant proteins in L-EV samples to glycolysis, gap junction, and metabolism, whereas those prevalent in S-EV samples were related to the lysosome, glycan degradation, and glycosaminoglycan biosynthesis (supplemental Table S7).

Finally, we compared the proteins quantified in pig sEVs with those included in the ExoCarta (http://www.exocarta.org/) and Vesiclepedia (http://www.microvesicles.org/) databases. When contrasted to the top 100 proteins identified as EVs markers in both databases, the overlap rates of our pig sEVs proteome were 64% and 65%, in the ExoCarta and Vesiclepedia proteome databases, respectively. In addition, 64 sEV proteins identified in the current study were not included in any EV database (supplemental Fig. S3).

## Discussion

A major handicap for EV research is the lack of a standardized isolation procedure to reliably isolate different EV subsets present in mammalian body fluids. The present study aimed to isolate two subsets of EVs, using procedures that included serial centrifugations as an initial step and a final centrifugation of 20,000*g*, which is considered adequate to separate small from large EVs, where the small ones remain in the supernatant and the large ones are captured in the pellet (43, 44, 45). The two isolated EV subsets were characterized in detail using a battery of complementary approaches following International Society of Extracellular Vesicles recommendations (MISEV 2018, (37)). Physical characterization, including morphology, size, and concentration, showed clear differences between EVs of the two subsets, although some size overlap was found between the two subsets. Such overlapping is, unfortunately, difficult to be avoided using current EV-isolation protocols (46). Furthermore, flow cytometry analysis revealed that EVs from both subsets showed a similar proportion of positivity to EV-specific protein markers. Overall, this characterization approach showed that each of the two sEV subsets was enriched in EVs exhibiting distinct physical characteristics.

Contamination with free proteins is a major concern in experiments aimed at deciphering the protein composition of EVs, implying that the isolation method has a significant influence on the extent of such contamination (47). In the present study, the sEV subsets were isolated using a SEC-based procedure combining serial centrifugations, SEC, and ultrafiltration. This procedure has been successfully used for EV isolation from a wide variety of biofluids (48), including semen (49), allowing for recovery of large amounts of EVs with a high degree of purity (50). The low presence of albumin, a high abundant protein in pig SP (51), in the recovered sEV samples would indicate that the EV samples handled in the present study have minimal cross-contamination by free proteins and, therefore, could be suitable for unraveling the proteome of EVs by MS-based proteomic analysis. Proteomic analyses backed this statement since albumin was one of the differentially abundant proteins between sEVs and non-EVs-enriched samples, being found in the highest amounts in non-EVs-enriched samples.

Extracellular vesicles contain a plethora of bioactive molecules, mainly proteins, which are delivered to target cells to elicit a specific response (52). In recent years, the improvement of MS-based techniques has made it possible to unravel the proteome of EVs circulating in several body fluids, leading to a better understanding of their functional roles in both physiological and pathological processes (53, 54). While the proteomic profile of sEVs has been well characterized in humans, identifying more than 3,500 proteins (3, 4, 6, 31, 55), high-throughput large-scale proteomic analysis of sEVs in other mammals is scarce, particularly in livestock. So far, Leahy *et al*. (5) and Rowlison *et al*. (56) carried out an in-depth proteomic analysis of sEVs, identifying a total of 520 and 3,008 proteins, in sheep and cats, respectively.

To the best of our knowledge, this is the first in-depth proteomic analysis of pig sEVs. Just a single, similar study was carried out in pigs identifying 28 sEV proteins using matrix-assisted laser desorption/ionization-TOF mass spectrometry technique (28). In the present study, a total of 988 proteins were identified and 737 of them were quantified by means of LC-MS/MS and SWATH-MS analysis, respectively. On analysis of the proteins identified in both studies, 28 *vs* 988, it is clear how the continuous improvement of MS-based techniques would allow for a better and more accurate identification of the EV proteome.

A comparison of the present proteome dataset of pig sEV with the previously published one for EVs from body fluids of other species and curated in Vesiclepedia and ExoCarta allowed us to identify 64 proteins in pig sEVs that have not previously been reported in EVs. Some of these “novel” EV proteins, such as the spermadhesins AQN-1, PSP-I, PSP-II, and AWN (51, 57), or the GPI-anchored protein TEX101 (58, 59), are relevantly involved in sperm function.

Growing evidence supports the notion that SP proteins are essential to modulate both sperm function and the female immune system (2). However, very little is known as to whether these modulating SP proteins solely circulate freely in the SP or are embedded in sEVs, where they might remain safe from SP proteases. To shed light on this issue, we have separately analyzed the proteome of two subsets of sEV, as well as the one from SP without EV. To the best of our knowledge, no other study has addressed the issue using a similar experimental approach or depth of scrutiny. Recently, Wang *et al*. (6) characterized the proteome of four human SP fractions resulting from density gradient ultracentrifugation, three with EVs and one without EVs. The latter, termed as “nonvesicular extracellular matter,” contained nonvesicular granular structures.

The proteome of each one of the two subsets of sEVs was compared with that of non-EVs-enriched samples. All quantified proteins were found in all three samples. This seemingly surprising finding could be explained by (1) circulating free proteins and vesicle-encapsulated proteins that were secreted by the same cells and/or (2) the inevitable cross-contamination between samples. To prevent this unavoidable interference from affecting the robustness of results, a highly discriminating cutoff criterion was used to identify differentially abundant proteins among samples. Thus, quantitative differences were found for several proteins between samples. The proteome of L-EVs was enriched in proteins exhibiting binding activity, mainly GTP binding. Among them, it is worth highlighting these belonging to small GTPases superfamily, including RAB10, RAB11B, RAB25, RALB, KRAS, RAP2C, RAP1B, RALA, RAC1, and RAN, which, in their active form (GTP bound), promotes vectorial membrane traffic (60). These proteins are involved in EV biogenesis and secretion (61, 62, 63). Other proteins enriched in L-EVs were VPS4B, an endosomal sorting complex required for transport of (ESCRT)-III associated protein involved in the endosomal multivesicular body pathway (64), and VAMP3 and STX3, two SNARE proteins involved in vesicular transport and membrane fusion (65, 66). The higher abundance in L-EVs of small GTPases, SNARE and ESCRT-III-associated proteins, could suggest that L-EVs were mainly secreted by the conventional secretion mechanisms of EVs, i.e., either by fusion of multivesicular bodies to the plasma membrane or by direct budding from the plasma membrane (67), rather than by the apocrine pathway, the secretory mechanism currently most widely accepted for seminal EV secretion, at least for S-EVs (21). Other proteins in higher abundance in L-EVs than in non-EVs-enriched samples were ACSL4, CLIC4, ELSPBP1, GPX4, PEBP1, SLC26A3, and SOD1, which GO analysis relate to reproductive processes. Some of these L-EV-enriched proteins could play a key role regulating sperm capacitation occurrence. Specifically, SLC26A3 (68), ELSPB1 (69) and PEBP1 (70) are some of which have been also identified in EVs isolated from bull (71) and ram (5) sEVs. Other two L-EVs-enriched proteins related to reproductive processes were antioxidant enzymes (GPX4 and SOD1), which were also identified in human sEVs by Zhang *et al*. (4). The presence of antioxidants has been well documented in EVs (72), including sEVs (73), which would be involved in minimizing sperm oxidative stress. Alvarez-Rodriguez *et al*. (74) reported that both SP enzymes were positively related to pig fertility, probably maintaining low levels of reactive oxygen species, for optimal sperm fertilizing capacity. In addition to these two antioxidant enzymes, other proteins with antioxidant properties such as CYBRD1, LDHA, LDHB, GSTp1, ATOX1, PRDX1, PRDX2, PRDX5, and ALDH9A1 were also found in higher abundance in L-EVs than in non-EVs-enriched samples. Spermatozoa are very sensitive to oxidative stress (75) and probably L-EVs, which are enriched with proteins with antioxidant activity, play a relevant role in minimizing sperm damage associated with oxidative stress. This suggestion would be supported by the findings of Du *et al*. (29) who demonstrated that sEVs were able to improve antioxidant capacity of pig spermatozoa, and by Saez *et al*. (73) and more recently by Wang (6), who have demonstrated that sEVs reduce the levels of reactive oxygen species in human spermatozoa.

Surprisingly, and in contrast to L-EVs, the proteome of S-EVs was quantitatively like that of non-EVs-enriched samples. This might indicate that the proteome of S-EV and non-EVs-enriched SP is different from that of L-EVs by the same extent as that discussed above between L-EVs and non-EVs-enriched SP. Indeed, 197 proteins were found in different amounts between L-EVs and S-EVs. The extent of proteome differences between both sEV subsets was similar to that found between EV subsets isolated in human (4, 6) and sheep (5) SP. Taken together, these results would support the view that several subsets of EVs coexist in SP, which show differences in cargo and, therefore, perhaps also in function, making it a challenge to isolate them to characterize their cargo and function.

Of the 197 proteins differentially abundant between the two subsets of EVs, 168 were in higher abundance in L-EVs than in S-EVs. As expected, most of these proteins were the same ones that were in higher abundance in L-EVs with respect to non-EVs-enriched SP, which have been discussed above. Since several of these differentially abundant proteins are involved in sperm functionality, we can assume that L-EVs would have a more relevant role than sEVs in key processes such as capacitation or by minimizing oxidative damage in spermatozoa. The proteome of S-EVs had 29 proteins in higher abundance than that of L-EVs, including cathepsins (CTSA, CTSC, CTSH, CTSF), GBA, PRCP, and LGMN, and the enzymes FUCA1, FUCA2, and LIPA; all of them are proteins linked to lysosome structure and function as revealed by GO enrichment analysis. It has been reported that exosomes could also be present in lysosomes, their content remaining protected from degradation, and further being released into the extracellular space through lysosomal pathways (76). In addition, it has also been reported they could be secreted through the apocrine pathway, i.e., embedded within apical blebs (21). Assuming these alternative secretion mechanisms, it is very plausible that the major secretion mechanism of S-EVs is the apocrine pathway rather than the conventionally accepted secretion mechanisms for the other body EVs, including L-EVs, as discussed above. It is worth mentioning that some of the proteins sEV-enriched are proteases (*i.e.*, cathepsins). These proteases could be localized on the surface of EVs and could be active in degrading substrates present in cells, including spermatozoa, and in the extracellular space (77). Other proteins in higher abundance in S-EVs than in L-EVs were CTSH and CTSC, both involved in immune-related processes, and GBA and GAS6, involved in regulating interleukin-6 production, as revealed by GO enrichment analysis. Thus, it is tempting to speculate that sEVs could contribute to the establishment of an optimal tolerogenic immune environment in the female genital tract, which is essential for successful sperm survival, conception, and embryo development (78). This would be supported by Bai *et al*. (30), who reported that pig sEVs were able to induce the expression of immune-related genes in *in vitro* cultured pig endometrial cells. The B4GALT1 was the only protein related to reproductive processes that was found in higher amounts in S-EVs than in L-EVs. This protein has been identified in mouse epididymosomes (79) and pig spermatozoa (80) and suggested to be involved in sperm–zona pellucida binding (81). This functional role of B4GALT1 could support the role of S-EVs in the fertilizing capacity of spermatozoa. Further functional studies should be carried out to confirm this hypothesis.

In conclusion, the present study demonstrates that the combination of differential centrifugations, ultrafiltration, and SEC provides a reliable methodology to isolate two pig seminal EV subsets, namely, small EVs and large EVs. Large-scale high-throughput proteomic analysis of these two sEV subsets allowed the identification and quantification of 737 proteins, 197 found differentially abundant. GO analysis of these suggest a distinct pattern of secretion of both subsets. Small sEVs could be mainly secreted following an apocrine pathway, carrying proteins related to promoting an appropriate immune environment in the female genital tract for optimal sperm and embryo survival, leading to successful pregnancy to term. Likewise, large sEVs could be secreted following conventional secretion of EVs, and their functional role would be more linked to sperm functionality, mainly by regulation of capacitation or minimizing oxidative stress.

## Data Availability

The mass spectrometry proteomics data have been deposited to the ProteomeXchange Consortium *via* the PRIDE (82) partner repository with the dataset identifier PXD039833. The annotated spectra were also deposited in Mendeley Data (https://data.mendeley.com/datasets/36mjr8r5c3/1). The data supporting the conclusions of this article will be made available by the authors, without undue reservation.

## Supplemental data

This article contains supplemental data.

## Conflict of interest

The authors declare no competing interests.

## Figures and Tables

**Supplementary Figure S3:**